# Role of Cr Doping on the Structure, Electronic Structure, and Electrochemical Properties of BiFeO_3_ Nanoparticles

**DOI:** 10.3390/ma15124118

**Published:** 2022-06-09

**Authors:** Shalendra Kumar, Faheem Ahmed, Naushad Ahmad, Nagih M. Shaalan, Rajesh Kumar, Adil Alshoaibi, Nishat Arshi, Saurabh Dalela, Mohammed Albossed, Keun Hwa Chae, Parvez Ahmad Alvi, Kavita Kumari

**Affiliations:** 1 Department of Physics, College of Science, King Faisal University, P.O. Box 400, Al-Ahsa 31982, Saudi Arabia; fahmed@kfu.edu.sa (F.A.); nmohammed@kfu.edu.sa (N.M.S.); adshoaibi@kfu.edu.sa (A.A.); malbossed@kfu.edu.sa (M.A.); 2 Department of Physics, University of Petroleum & Energy Studies, Dehradun 248007, India; 3 Department of Chemistry, College of Science, King Saud University, P.O. Box 2455, Riyadh 11451, Saudi Arabia; anaushad@ksu.edu.sa; 4 Physics Department, Faculty of Science, Assiut University, Assiut 71516, Egypt; 5 University School of Basic and Applied Sciences, Guru Gobind Singh Indraprastha University, New Delhi 110078, India; rajeshpositron@gmail.com; 6 Department of Basic Sciences, Preparatory Year Deanship, King Faisal University, P.O. Box 400, Hofuf Al-Ahsa 31982, Saudi Arabia; nshastri@kfu.edu.sa; 7 Department of Pure & Applied Physics, University of Kota, Kota 324005, India; sdphysics@rediffmail.com; 8 Advanced Analysis Center, Korea Institute of Science and Technology, Seoul 136-791, Korea; khchae@kist.re.kr; 9 Department of Physics, Banasthali Vidyapith, Banasthali 304022, India; parveznihal@gmail.com; 10 School of Materials Science and Engineering, Changwon National University, Changwon 51140, Korea; kkmalhan@gmail.com

**Keywords:** Cr doped BiFeO_3_, supercapacitor, electrochemical, ferromagnetic, band gap

## Abstract

BiFe_1−x_Cr_x_O_3,_ (0 ≤ x ≤ 10) nanoparticles were prepared through the sol–gel technique. The synthesized nanoparticles were characterized using various techniques, viz., X-ray diffraction, high-resolution field emission scanning electron microscopy (HRFESEM), energy dispersive spectroscopy (EDS), UV–Vis absorption spectroscopy, photoluminescence (PL), dc magnetization, near-edge X-ray absorption spectroscopy (NEXAFS) and cyclic voltammetry (CV) measurements, to investigate the structural, morphological, optical, magnetic and electrochemical properties. The structural analysis showed the formation of BiFeO_3_ with rhombohedral (R3c) as the primary phase and Bi_25_FeO_39_ as the secondary phase. The secondary phase percentage was found to reduce with increasing Cr content, along with reductions in crystallite sizes, lattice parameters and enhancement in strain. Nearly spherical shape morphology was observed via HRFESEM with Bi, Fe, Cr and O as the major contributing elements. The bandgap reduced from 1.91 to 1.74 eV with the increase in Cr concentration, and PL spectra revealed emissions in violet, blue and green regions. The investigation of magnetic field (H)-dependent magnetization (M) indicated a significant effect of Cr substitution on the magnetic properties of the nanoparticles. The ferromagnetic character of the samples was found to increase with the increase in the Cr concentration and the increase in the saturation magnetization. The Fe (+3/+4) was dissolved in mixed-valence states, as found through NEXAFS analysis. Electrochemical studies showed that 5%-Cr-doped BFO electrode demonstrated outstanding performance for supercapacitors through a specific capacitance of 421 F g^−1^ measured with a scan rate of 10 mV s^−1^. It also demonstrated remarkable cyclic stability through capacitance retention of >78% for 2000 cycles.

## 1. Introduction

Electrochemical energy storage devices, including batteries, fuel cells and supercapacitors, adequately meet the constantly increasing demands of portable energy devices. Recently, supercapacitors have become a significant area of interest for the research community due to their high specific capacitance and remarkable cyclic stability [1,2,3,4,5]. The supercapacitor electrodes combine the faradaic redox reaction and the non-faradaic electrostatic charge storage mechanisms and provide fast ion diffusion and a high specific surface area, which helps to achieve high energy density. Researchers have been working on the development of an electrode material which can involve both rapid charging/discharging of supercapacitors and high energy density of the batteries. The performance of the supercapacitor can be enhanced by increasing the energy density and electrochemical cycling stability, leading to highly efficient electrode materials [6,7]. The measurement of the supercapacitance performance of the electrode material is performed by cyclic voltammetry (CV) galvanostatic charging–discharging. The electrode material should be fabricated using a material with high specific capacitance and a large number of charge–discharge cycles [8]. For this purpose, bismuth ferrite (BFO) has emerged as a promising material owing to its modifiable redox properties and charge storage capacity [9,10,11,12,13,14,15]. Devi et al. have well described the energy storage applications of the Bi-based materials, including Li-ion batteries, fuel cells, etc. [6].

Moreover, in the last few decades, multiferroic materials have gained the great attention of the scientific community due to their advanced technological applications [16,17,18,19,20,21]. Among them, BFO also possesses vital coexisting multiferroic properties such as ferromagnetism and ferroelectricity. The distorted rhombohedral crystal structure of BFO allows deformation in the central-local symmetry due to the spontaneous polarization caused by 6s^2^ lone pairs of Bi^3+^ ions which give rise to the ferroelectric properties of the material. On the other hand, the exchange interactions involving Fe^3+^ ions result in the ferromagnetic ordering [22]. BFO demonstrates a ferromagnetic curie temperature of ~1102 K and an antiferromagnetic N$\overset{´}{e}$el temperature of ~644 K. There are many existing reports which show significant multiferroic properties in BFO [23]. Interestingly, both the properties function simultaneously, showing a co-dependent magnetoelectric relationship. Due to these remarkable properties, BFO has been established as a potential candidate for technological applications in the fields of spintronics and communications. However, the synthesis of single-phase BFO is very challenging because of the formation of secondary phases along with the BiFeO_3_ phase. Generally, the BiFeO_3_ phase is accompanied by secondary phases—Bi_2_Fe_4_O_9_, Bi_25_FeO_39_, etc. [8,24,25]. Suppressing the secondary phases is a quite challenging task. Even so, the values of the high specific capacitance make BFO an important candidate for electrochemical applications. The formation of a single phase and the enhancement in specific capacitance of BFO can be accomplished by the doping of rare earth or transitions metal elements at the A site or B site, respectively. The doping of foreign elements at the position of the host cation at low concentrations can work wonders when it comes to reducing the secondary phases and enhancing the values of specific capacitance. Recently, Kumar et al. have obtained a specific capacitance of 328 F/g in La-doped BFO, resulting in an excellent supercapacitor performance [3]. For this reason, BFO has been highly explored lately for obtaining an electrode material with enhanced supercapacitance performance.

Therefore, in the present work, we have prepared BiFe_1−x_Cr_x_O_3,_ (0 ≤ x ≤ 10) nanoparticles via sol–gel chemical route. The synthesized nanoparticles were characterized via various characterization techniques, viz., X-ray diffraction, high-resolution field emission scanning electron microscopy (HRFESEM), energy dispersive spectroscopy (EDS), UV–vis absorption spectroscopy, photoluminescence (PL), dc magnetization, near-edge X-ray absorption spectroscopy (NEXAFS) and electrochemical measurements to investigate the structural, morphological, optical and magnetic properties and electrochemical performance.

## 2. Experimental Details

### 2.1. Materials

Bismuth nitrate pentahydrate [Bi(NO_3_)_3_·5H_2_O]; iron nitrate nonahydrate [Fe (NO_3_)_3_·9H_2_O]; chromium nitrate hexahydrate [Cr(NO_3_)_3_·6H_2_O], citric acid (C_6_H_8_O_7_), concentrated nitric acid (HNO_3_) and ethylene glycol. All precursor salts and reagents were of high purity (>97%) and purchased from Central Drug House, (CDH, New Delhi, India).

### 2.2. Method

BiFe_1−x_Cr_x_O_3,_ (0 ≤ x ≤ 10) nanoparticles were synthesized using the sol–gel method. An appropriate amount of citric acid (C_6_H_8_O_7_) was dissolved in 50 mL of DI water to obtain a molar concentration of 0.1 M. The ratio of metal nitrate and citric acid was kept at 1:1. The mixture was stirred at room temperature to make a solvent for further reaction. Then, Bi(NO_3_)_3_·5H_2_O was added to the solvent, followed by an appropriate amount of concentrated HNO_3_ to dissolve Bi(NO_3_)_3_·5H_2_O. Once Bi(NO_3_)_3_·5H_2_O was dissolved, chromium nitrate hexahydrate [Cr (NO_3_)_3_·6H_2_O] and iron nitrate nonahydrate (Fe(NO_3_)_3_·9H_2_O) were added as per their pre-decided ratio. These precursor salts were mixed well at room temperature. Finally, ethylene glycol was added to the mixture under vigorous stirring at 90 °C to form a gel and allowed to dry completely. The obtained product was grinded well and calcined at 550 °C for 2 h.

### 2.3. Sample Characterizations

The synthesized products were characterized using X-ray diffraction, high-resolution field emission scanning electron microscopy (HRFESEM), energy dispersive spectroscopy (EDS), UV–vis absorption spectroscopy, photoluminescence (PL) and dc magnetization. A Bruker AXS D8 X-ray diffractometer with Cu Kα radiation (λ = 1.54178 Å; 2θ range: 10°–90°), at a scanning rate of 0.02°/s, was used to study the phase purity of the samples. The HRFESEM micrographs and EDS spectra were obtained with a field emission electron microscope (FESEM, JSM-7500, JEOL, Tokyo, Japan). The optical properties were studied using UV–vis absorption spectroscopy performed using a photo-spectrometer (S-4100) from SINCO Instrument Co. Seoul, Korea. The PL spectra were obtained at Model LAMBDA 35, PerkinElmer (Waltham, MA, USA). The M-H hysteresis loop measurements were carried out using Quantum Design physical properties measurement setup (PPMS-6000, Quantum Design, San Diego, CA, USA). The Fe L_3,2_ and O K-edge spectra were measured with the soft X-ray beamline 10D XAS KIST (Korea Institute of Science and Technology) of Pohang Accelerator Laboratory (PAL). A typical three-electrode experimental cell of an electrochemical analyzer (Corrtest-CS150, Wuhan, China) was used to perform the electrochemical measurements of Cr doped BiFeO_3_ nanoparticles. The pretreated nickel foam of the size 2 cm × 1 cm was used to prepare the electrode. The mechanism of the electrode fabrication was as follows: Pure and Cr doped BiFeO_3_ in different concentrations, polyvinylidene fluoride (PVDF) and carbon black were mixed in the ratio of 80:10:10. All the chemicals were mixed homogeneously using n-methyl-2 pyrrolidinone (NMP) as a solvent to form a slurry and then coated onto the nickel foam. Finally, the nickel foam was kept for drying at 80 °C in the oven for 24 h. All the experiments were performed in a 2 M KOH aqueous solution as an electrolyte, at room temperature, with Ag/AgCl as a reference electrode and Pt wire as a counter electrode. The electrochemical property of Cr doped BiFeO_3_ was examined with cyclic voltammetry (*CV*). The *CV* measurements were in the range of 0.0 to 0.6 V. The specific capacitances (*C*, (F/g)) of all the studied samples were determined using the relation [3]:

(1) $$C=\frac{1}{2mVk}\int_{V^{-}}^{V^{+}}I\left(V\right)dV\;\;By\;CV$$

where *C* (F g^−1^) defines the specific capacitance, *I* (A) represents discharge current, *V* (V) corresponds to the potential range, m (g) represents the mass of the active material and k (V s^−1^) is scan rate. Electrochemical impedance spectroscopies (EIS) measurements were carried out between 1 and 100 MHz using a sinusoidal perturbation of amplitude 10 mV.

## 3. Results and Discussion

### 3.1. XRD Analysis

**Figure 1:**
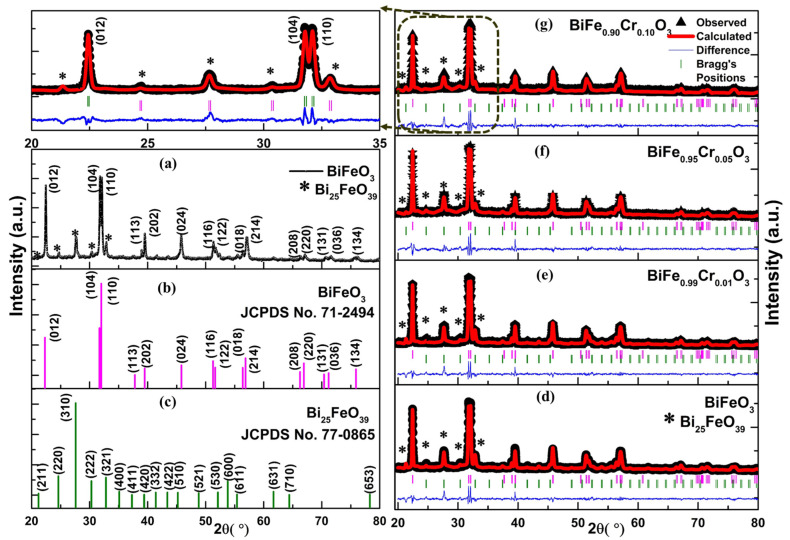
(**a**) XRD patterns of the undoped BiFeO_3_ showing the peak positions and * represent the secondary phase of Bi_25_FeO_39_. (**b**,**c**) JCPDS card numbers indicating peaks for BiFeO_3_ and Bi_25_FeO_39_, respectively. (**d**–**g**) The Rietveld refinement of the theoretically calculated patterns (red) superimposing the experimental data (black) for BiFe_1−x_Cr_x_O_3_ (0 ≤ x ≤ 10) nanoparticles. The difference between the two is indicated by the blue line at the bottom; the Bragg positions for BiFeO_3_ phase are indicated by the pink vertical lines; the Bragg positions for the secondary phase (Bi_25_FeO_39_) are indicated by the vertical green lines.

The identification of the crystalline phases present in the nanoparticles and variations in the structural parameters as a consequence of Cr doping in BFO was performed by investigating the X-ray diffraction patterns. Figure 1a demonstrates the XRD pattern of undoped BFO obtained experimentally. This pattern shows the sharp peaks indicating the polycrystalline nature of the nanoparticles. The initial identification of the peaks was performed by matching with the standard peak positions corresponding to the JCPDS card numbers: 71-2494 for pure BiFeO_3_ and 77-0865 for Bi_25_FeO_39_, as displayed in Figure 1b,c [24]. This reveals the presence of two phases in the samples: one, BiFeO_3_ as the primary phase, and the second, Bi_25_FeO_39_ as the secondary. BiFeO_3_ possesses rhombohedral symmetry with the R3c space group, whereas Bi_25_FeO_39_ possesses cubic symmetry with the I23 space group [25]. The absence of peaks corresponding to Cr shows that Cr ions are good substitutes for the Fe ions in the BFO lattice. Further, the Rietveld refinement was performed using the FULLPROF program [26] to ascertain the existence of these phases. The refined patterns of BiFe_0.90_Cr_0.10_O_3_ are displayed in Figure 1d–g. Two-phase refinement was carried out using R3c and I23 space groups. The black lines represent the experimental data points, the overlapping red lines indicate the theoretically matched patterns and the blue line at the bottom indicates the difference between the two. Bragg’s positions of the reflecting places are indicated by the vertical lines below the peaks: the pink vertical lines indicate peak positions of the primary phase BiFeO_3_, and the green vertical lines indicate the peak positions of the secondary phase Bi_25_FeO_39_. The major peaks corresponding to the secondary phase are marked in the patterns. The theoretically obtained patterns agree well with the experimental data points, showing good-quality fitting. Further, the refinement process was assessed quantitatively by the values of the reliability factors and χ^2^ [27]. The values of the reliability factors and the phase fractions of both phases are indicated in Table 1. The effect of Cr doping can be clearly seen in the phase fractions. As the Cr concentration increases in the nanoparticles, the fraction of the primary phase increases and that of the secondary phase decreases. This points toward the fact that an increase in Cr content also increases the dilution limit of the primary phase resulting into suppression of secondary phase.

**Figure 2:**
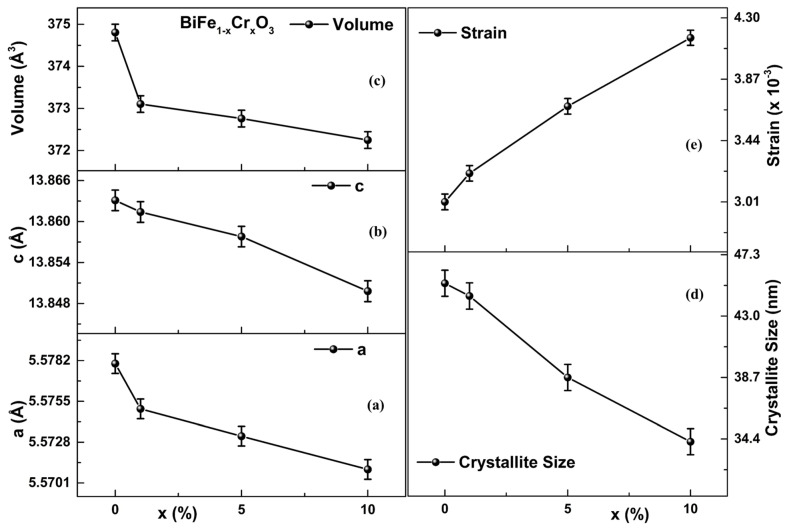
(**a**–**e**) Variations of lattice parameters (**a**,**c**), unit cell volume, crystallite size and strain as functions of Cr concentration.

The structural parameters as functions of the Cr concentration are represented in Figure 2a–e. The value of lattice parameters (a/c) for undoped BiFeO_3_, obtained from Rietveld refinement, was 5.5780/13.8631 Å, corresponding to a unit cell volume of 374.8 Å^3^ [28]. These values of lattice parameters are in agreement with the literature [29,30]. Both the lattice parameters (“a” and “c”) were found to decrease along with the unit cell volume as Cr concentration increased, as displayed in Figure 2a–c. Likewise, the crystallite sizes calculated using Scherrer’s formula were also found to decrease with increasing Cr concentration from 45.0 nm (BiFeO_3_) to 34.0 nm (BiFe_0.90_Cr_0.10_O_3_), as can be seen in Figure 2d. Similar results have been reported by Layek et al.: they found that the minimum crystallite size was 39 nm for BiFe_0.90_Cr_0.10_O_3_ [31]. The strain calculated using Wilson’s formula [32] was found to increase with increasing Cr concentration (Figure 2e). A possible reason is that the ionic radius of Cr^3+^ is smaller than that of Fe^3+^ (0.645 Å), due to which the substitution of Cr^3+^ (0.615 Å) in place of Fe may reduce the crystallite size and lattice parameter. Owing to this, reductions in the structural parameters have been found to increase with the increase in the Cr content in the samples. Further, these reductions in the structural parameters may be the possible cause of the strain developed in the lattice.

### 3.2. Morphological Analysis

**Figure 3:**
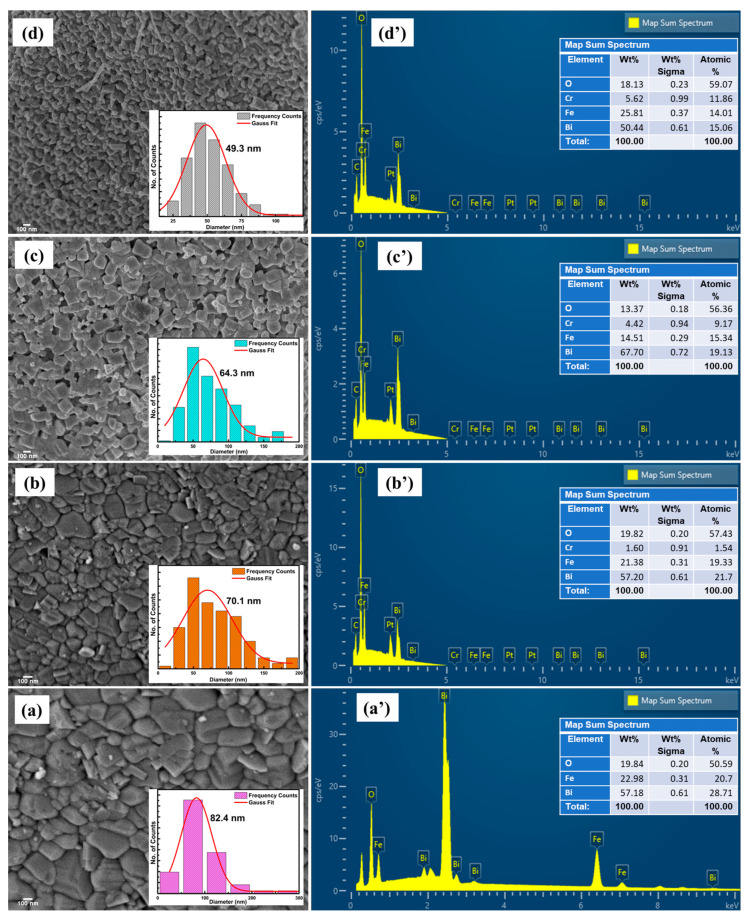
(**a**–**d**) HR-FESEM micrographs of BiFe_1−x_Cr_x_O_3_ (0 ≤ x ≤ 10) nanoparticles; insets show the histograms of particle size distribution. (**a**’–**d**’) EDS spectra of BiFe_1−x_Cr_x_O_3_ (0 ≤ x ≤ 10) nanoparticles; insets show the wt % and at% of the respective compositions.

The morphological and compositional analysis were performed through the investigation of high-resolution field emission scanning microscopy and energy-dispersive X-ray spectroscopy, respectively. The HR-FESEM micrographs of BiFe_1−x_Cr_x_O_3_ (0 ≤ x ≤ 10) nanoparticles are displayed in Figure 3a–d. The micrographs demonstrate a broad distribution of morphologies. The size distribution histograms are represented in the insets of the respective figures. The average particle size was found to decrease with increasing Cr in the samples. The spectra of the samples are displayed in Figure 3a’–d’. The spectra display the major contributions of Fe, Bi, O and Cr. The wt % and at% of the composition are displayed in the insets of Figure 3a’–d’. The compositions of the compounds were in accordance with the doping.

### 3.3. UV–Vis Spectroscopy

**Figure 4:**
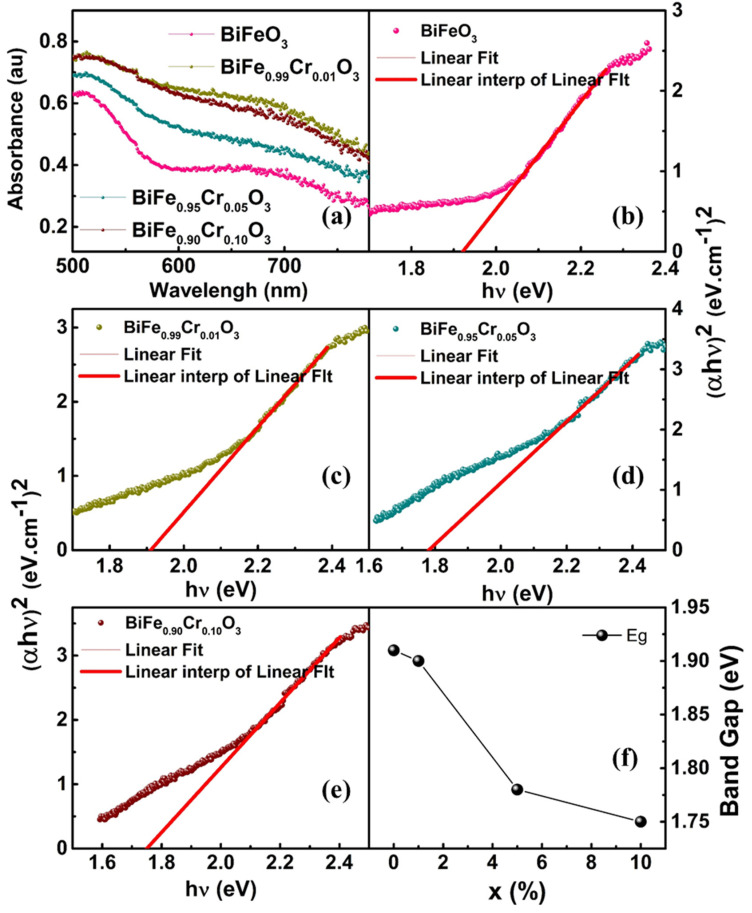
(**a**) UV–Vis absorption spectra. (**b**–**e**) Tauc’s plots indicating interpolated fitted linear region and (**f**) band gaps of BiFe_1−x_Cr_x_O_3_ (0 ≤ x ≤ 10) nanoparticles as a function of Cr concentration.

Figure 4a shows the UV–Vis absorption spectra of BiFe_1−x_Cr_x_O_3_ (0 ≤ x ≤ 10) nanoparticles in the wavelength range 500–800 nm. The maximum absorption was observed at ~510.0 nm and decreased afterward at higher wavelengths. The bands observed at maximum absorption may have arisen due to the direct charge transfer excitation involving *d-d* transitions between neighboring Fe^3+^ ions [33]. The values of the bandgap energies have been calculated using the Tauc’s equation, *(αhν)^2^ = B (hν − E_g_)*, where *α*: absorption coefficient, *h*: Planck’s constant, *hν*: absorbed photon energy and *B*: the constant. The Tauc’s plots of the samples are presented in Figure 4b–e. The linear regions of the plots at higher frequencies were linearly fitted and extrapolated, and intersected at the x-axis at zero absorption. The point of intersection gives the value of the bandgap. The bandgap energies were found to decrease with increasing Cr concentration. The value of band gaps varied from 1.91 eV for undoped BFO to 1.74 eV for BiFe_0.90_Cr_0.10_O_3_ nanoparticles, as represented in Figure 4f. The highest value so obtained was 1.91 eV for undoped BFO.

### 3.4. Photoluminescence (PL) Spectroscopy

**Figure 5:**
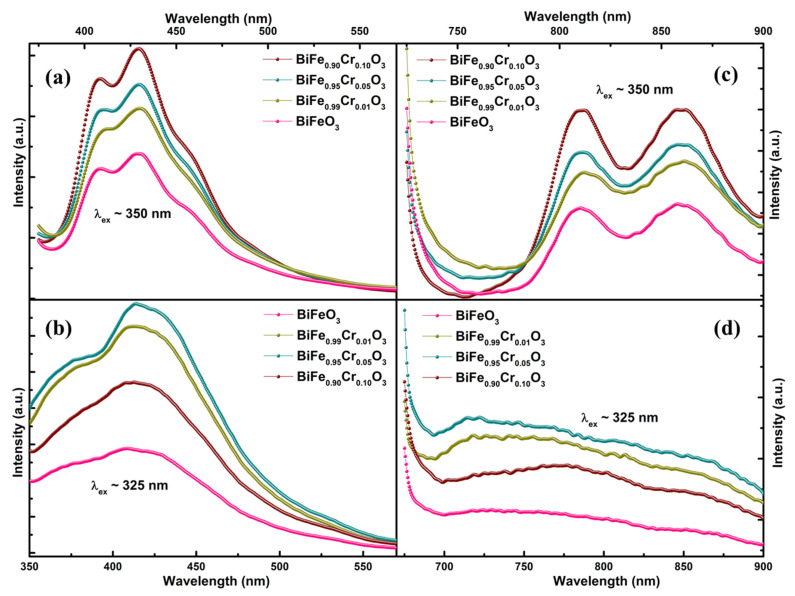
P–L spectra of BiFe_1−x_Cr_x_O_3_ (0 ≤ x ≤ 10) nanoparticles in the ranges (**a**,**b**) 300–600 nm and (**c**,**d**) 600–900 nm with the excitation wavelengths of 350 and 325 nm.

**Figure 6:**
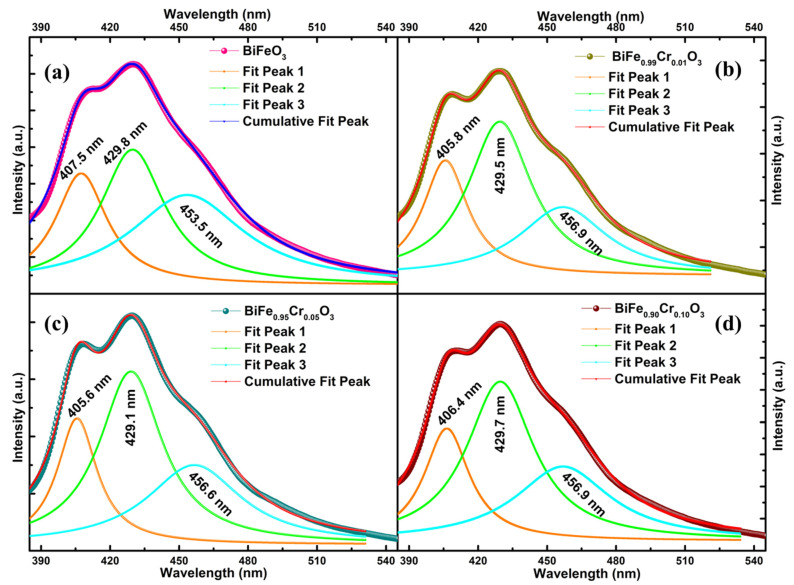
(**a**–**d**) Fitting of P–L spectra of BiFe_1−x_Cr_x_O_3_ (0 ≤ x ≤ 10) nanoparticles in the range 300–600 nm with Lorentzian peak function.

**Figure 7:**
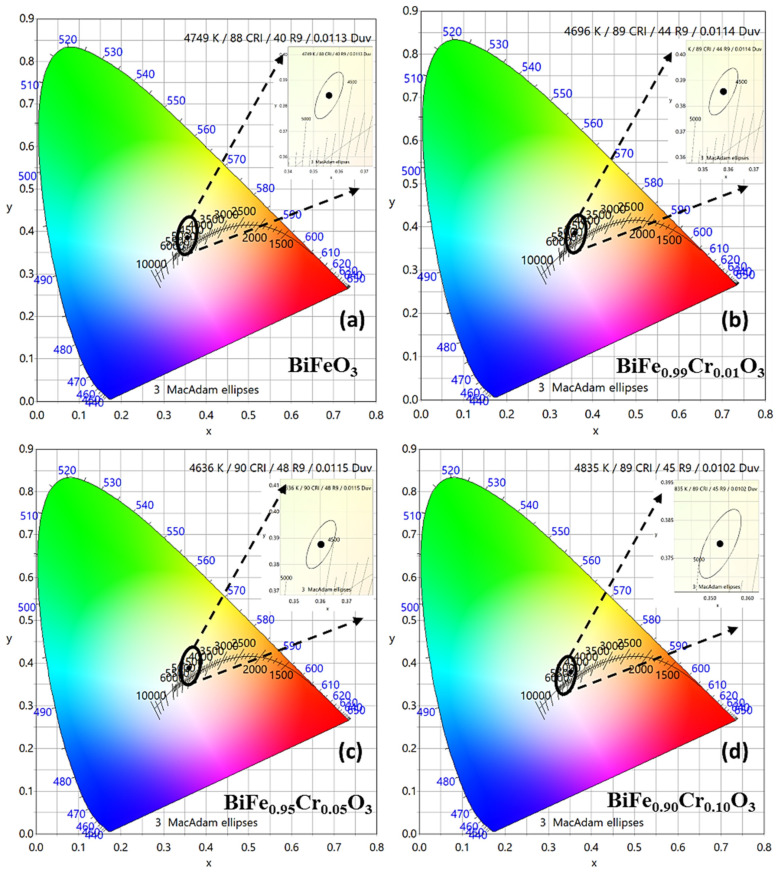
(**a**–**d**) CIE 1931 chromaticity diagram of BiFe_1−x_Cr_x_O_3_ (0 < x < 10) nanoparticles in the range 300–700 nm; respective insets show the enlarged views of the emitted colors shown by the elliptical dots.

The band structure and the defect states were further investigated via PL spectroscopy. Figure 5a–d demonstrates the normalized PL response of BiFe_1−x_Cr_x_O_3_ (0 ≤ x ≤ 10) nanoparticles in the low (~300–600 nm) and high (~700–900 nm) wavelength ranges. Figure 5a,b and Figure 5c,d represent the spectra measured by illuminating the nanoparticles with the excitation wavelengths (λ_ex_) of 350 nm (~3.54 eV) and 325 nm (~3.82 eV), respectively. The differences between the spectra measured at different excitation wavelengths are absolutely evident from the spectra. Figure 5a demonstrates the prominent bands in the range 400–480 nm corresponding to the sharp emissions in violet and blue regimes [34]. A negligibly small band also appears around 495 nm, indicating trivial emissions in the green region. Strong bands in BFO generally arise due to the number of transitions involving band to band, near-band and defect state emissions. These bands corresponding to the involved transitions give information about the recombination centers, which can further give information about the defect states present in the material [34]. The samples exhibited all these bands identically irrespective of the concentration of Cr, suggesting the absence of any defect states induced due to doping. However, the intensity was found to increase with the increasing Cr concentration. The intensity of the PL spectra shows the strength of the electronic transition and is directly related to the recombination of the charge carriers [35]. That means, greater the recombination, greater is the intensity of the PL emission. Therefore, the increasing intensity with increasing Cr concentration in the present case indicates a higher amount of recombination. This may be correlated with the narrowing of the band gaps with increasing Cr concentration, which was revealed in the investigation of UV–Vis absorption spectra in Section 3.3. The highly sharp bands were previously observed by Guo et al., measured with an excitation wavelength of 405 nm, corresponding to the blue emission [36]. When these bands (λ_ex_~350 nm) are compared with the bands measured with the λ_ex_~325 nm, as can be observed in Figure 5b, the distinction between the bands disappears, resulting in a single broad band associated with the blue emission, and the violet emissions are suppressed. This indicates that the excitation wavelength with corresponding energy significantly influences the emission by selecting the electrons specifically for excitation in the material. Similar behavior has also been indicated by the spectra obtained in the near infrared region. Two main bands can be observed which are similar for all the samples for λ_ex_~350 nm, as shown in Figure 5c. These bands indicate the emissions in red-infrared regions [37]. However, no sharp bands can be observed for λ_ex_~325 nm in Figure 5d. Furthermore, the deconvoluted bands with Lorentzian curve fitting in the low wavelength region are displayed in Figure 6a–d for BiFe_1−x_Cr_x_O_3_ (0 ≤ x ≤ 10) nanoparticles. The deconvolution of bands reveals three main bands in the violet–blue regime. Although the shift in wavelength with respect to Cr concentration is negligible, this variation is an indication of the band energies. Thus, the nanoparticles altogether illustrated prominent emissions in violet, blue and trivial green regimes corresponding to an excitation wavelength of 350 nm and no observable optical transitions. However, the emissions were suppressed as a consequence of the reduced excitation wavelength of 325 nm. In addition, the Commission Internationale de I’Eclairage (CIE) color coordinates are represented in CIE chromaticity diagrams, as displayed in Figure 7a–d in x,y space. The color points are indicated in enlarged views displayed in the insets of Figure 7a–d. The obtained parameters are indicated in Table 2. The color points depicted by the x,y coordinates demonstrate the light emission in a region which is neither towards the boundaries nor at the exact center. That means the emitted light does not possess a pure color, nor is it pure sunlight. Emission was demonstrated in the light green, yellow and violet regions and had only slight variations with the dopant concentration.

### 3.5. Magnetization Analysis

**Figure 8:**
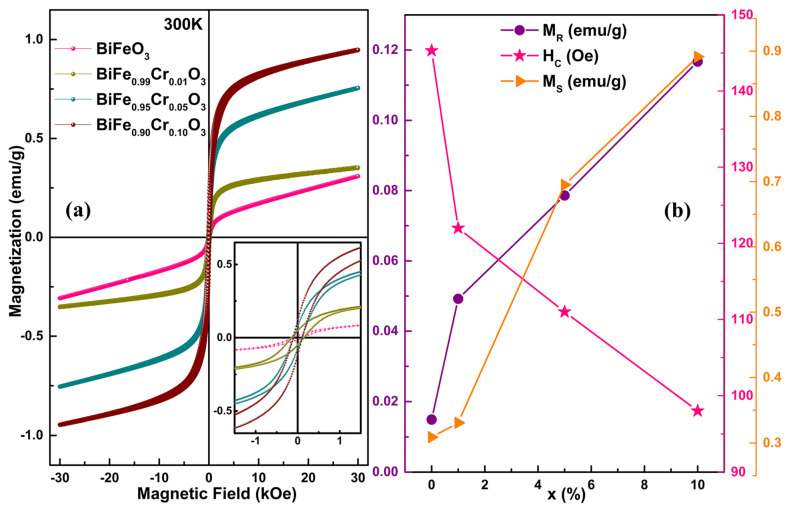
(**a**) Room temperature M–H hysteresis curves of BiFe_1-x_Cr_x_O_3_ (0 ≤ x ≤ 10) nanoparticles in the range ±30 kOe; inset shows the behavior of the curves near H = 0. (**b**) Variation in M_R_, Ms and Hc as functions of Cr concentration.

The room temperature magnetic hysteresis curves, measured at an applied magnetic field (H) of ±30 kOe, for BiFe_1−x_Cr_x_O_3_ (0 ≤ x ≤ 10) nanoparticles, are displayed in Figure 8a. All the samples show hysteresis; however, the maximum coercivity (H_C_) was observed for undoped BFO only, which was reduced after doping with Cr. The coercivity was found to be 145 Oe for BiFeO_3_ and 98 Oe for BiFe_0.90_Cr_0.10_O_3_ nanoparticles, as demonstrated in Figure 8b. The decrease in coercivity may have been associated with the decreasing magnetic anisotropy in the nanoparticles [38]. The effect of Cr concentration on the magnetization (M) is quite evident, showing the increase in magnetization with an increase in Cr concentration. Although all the samples exhibited room-temperature ferromagnetic behavior, as displayed by the inset of Figure 8a, saturation was not attained at ±30 kOe. This may have been due to the G-Type antiferromagnetic nature originally exhibited by BFO. The ferromagnetic behavior shown by undoped BFO may have been due to its reduced crystallite dimensions resulting in the distortion of its crystalline structure. Sinha et al. have also reported that the reduction of crystalline dimensions below 62.0 nm brings ferromagnetic character in the material; otherwise, BFO is antiferromagnetic [38]. Therefore, to obtain saturation magnetization (M_S_), 1/M Vs H were plotted. The values of M_S_ are displayed in Figure 8b. Unlike H_C_, M_S_ was found to increase from 0.31 emu/g for BiFeO_3_ to 0.98 emu/g for BiFe_0.90_Cr_0.10_O_3_ with increasing Cr concentration. Similarly, remnant magnetization (M_R_) was also found to increase with increasing Cr concentration from 0.01 emu/g to 0.1 emu/g. These values of the magnetization parameters are comparable with other reported values [39]. Kharel et al. have also reported the enhancement in the magnetic properties of Cr doped BiFeO_3_ [40]. The enhancement in the magnetization of the nanoparticles as a result of increasing Cr concentration may be associated with the strong magnetic interactions caused by Fe^3+^–O–Cr^3+^ coupling. The substitution of Cr^3+^ ions in place of Fe^3+^ ions leads to strong super-exchange interactions which significantly influence the magnetic parameters by causing structural distortions [36,41]. Moreover, the enhancement in magnetization may also be associated with the secondary phase Bi_25_FeO_39_ [38]. However, this should not be the reason in present case because of the reduction of secondary phase fraction with increasing Cr concentration. Therefore, it can be concluded that increasing the doping concentration of Cr in BiFeO_3_ enhances the magnetic properties of the material due to the enhanced Fe^3+^–O–Cr^3+^ interactions.

### 3.6. Near Edge X-Ray Absorption Spectroscopy

#### 3.6.1. Fe L_3,2_ Edge

**Figure 9:**
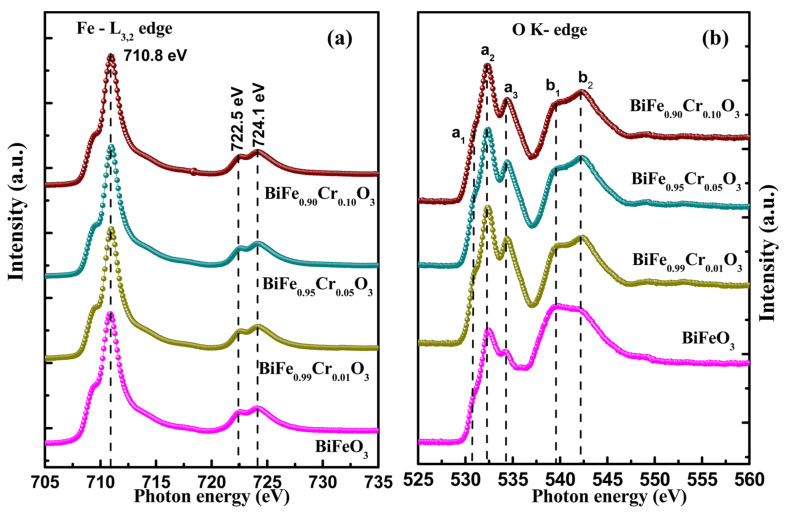
(**a**) Fe L_3,2_ edge and (**b**) O K edge spectra of BiFe_1−x_Cr_x_O_3_ (0 ≤ x ≤ 10) nanoparticles.

The Fe L_3,2_ edge spectra of BiFe_1−x_Cr_x_O_3_ (0 ≤ x ≤ 10) nanoparticles is displayed in Figure 9a in the range 705–735 eV. The L-edge spectra arise as a result of the transitions between Fe *2p* and Fe *3d* states. Since the spectra have resulted from the core electronic transitions among Fe *2p_3/2_* and Fe *2p_1/2_* states, they provide evidence of the unoccupied states of the probed element. The L-edge spectra were further split into L_3_ and L_2_ because the neighboring spin-up and spin-down states experience strong exchange interactions. The highest intensity peak (L_3_-edge) was observed at 710.8 eV with a small shoulder on the right side. The other peak (L_2_-edge) observed at higher energies shows peak splitting of ~1.6 eV, indicating peaks at 722.5 and 724.1 eV. The small shoulder peak on the right side of the L_3_-edge and the splitting in the L_2_ edge may be associated with the crystal field effects caused by the octahedral structural symmetry. No detectable shift in the edge positions was obtained due to varying Cr concentration. This reveals that the ionic state remains the same irrespective of the Cr concentration. By comparing these with the NEXAFS spectra of (Fe^2+^)O, (Fe^3+^)_2_O_3_ and (Fe^+3^/^+4^)_3_O_4_ [3], the Fe in the present samples was confirmed to be in the mixed-valence state.

#### 3.6.2. O K Edge

The O K edge spectrum of BiFe_1−x_Cr_x_O_3_ (0 ≤ x ≤ 10) nanoparticles is displayed in Figure 9b for the range 525–560 eV. The O K edge spectra involve a transition from O *2p* core states to higher-order states. The peaks observed at 530.5 eV (a_1_) and 532.3 eV (a_2_) may be attributed to the hybridization between the O *2p* and Fe *3d* states, and the peak observed at 534.2 eV (a_3_) may be attributed to the hybridization between O *2p* and Bi *6sp* states. The intensity of these peaks indicates the density of unoccupied highly states [42,43]. Another set of peaks (b_1_ and b_2_) with comparatively smaller intensities is present at higher energies. The origin of these peaks has been associated with the hybridization between O *2p* and Fe *4s* and Fe *4p* states. The intensities of b_1_ and b_2_ disclose the charge transfer phenomena occurring from O *2p* to its ligand states [44,45,46].

### 3.7. Electrochemical Analysis

**Figure 10:**
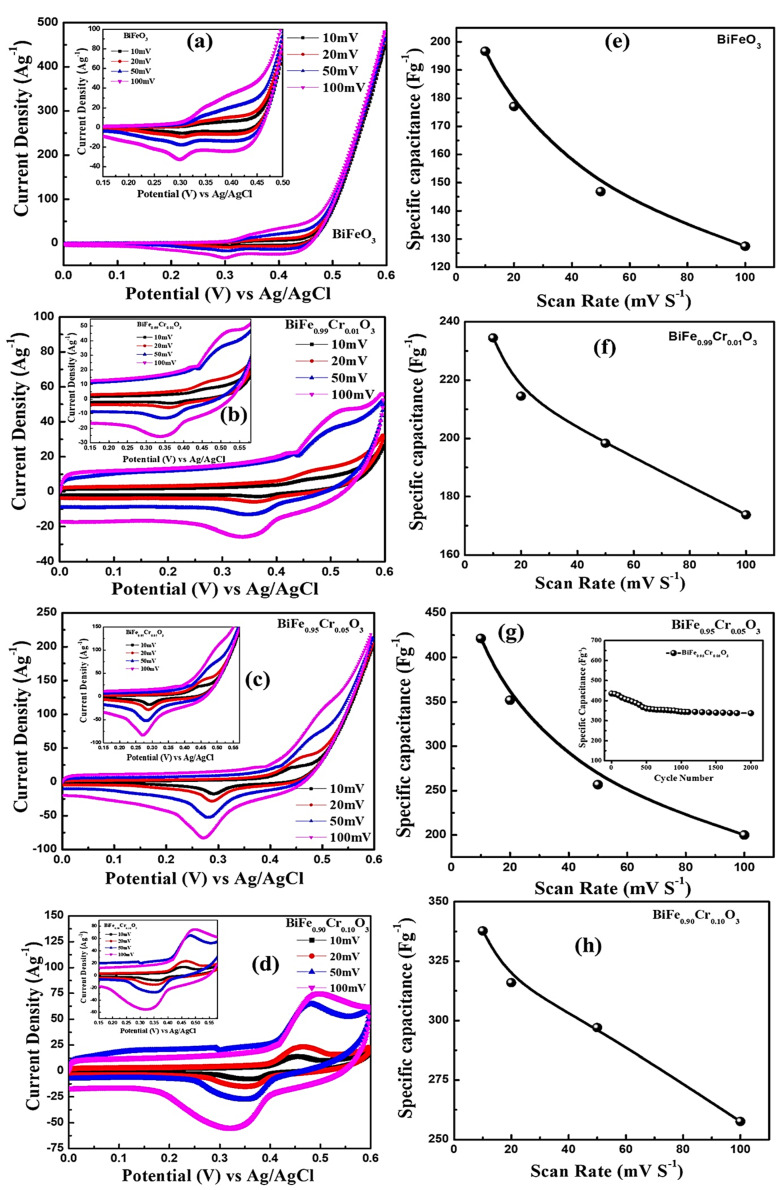
(**a**–**d**) CV plots of BiFe_1−x_Cr_x_O_3_ (0 ≤ x ≤ 10) nanoparticles with different scan rates. (**e**–**h**) Variation in the specific capacitance of the BiFe_1−x_Cr_x_O_3_ (0 ≤ x ≤ 10) nanoparticles electrode with different scan rates.

**Figure 11:**
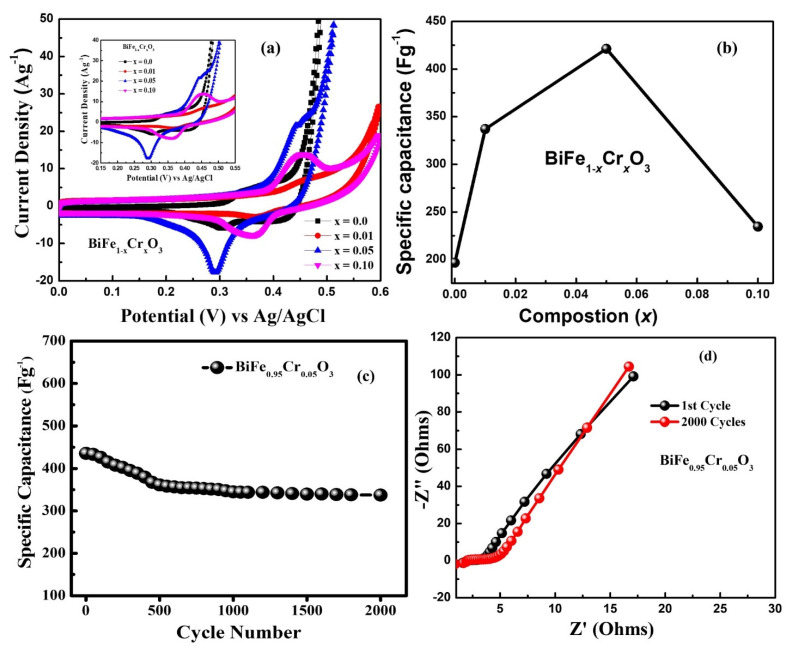
(**a**) CV plots of BiFe_1-x_Cr_x_O_3_ (0 ≤ x ≤ 10) nanoparticles at a scan rate of 10 mV S^−1^. (**b**) Variation in specific capacitance of BiFe_1−x_Cr_x_O_3_ (0 ≤ x ≤ 10) nanoparticles. (**c**) Cyclic performance of BiFe_0.95_Cr_0.05_O_3_ electrode for 2000 cycles. (**d**) Nyquist plots of BiFe_0.95_Cr_0.05_O_3_ electrode for 1st cycle and 2000th cycle.

The electrochemical performances of BiFe_1−*x*_Cr*_x_*O_3_ (0 ≤ x ≤ 10) electrodes were studied with the help of the cyclic voltammetry (CV). The CV curves of all the electrodes were recorded in a 2 M KOH aqueous solution with the three-electrode system in the range of 0.0 to 0.6 V at different potential scan rates of 10–100 mV s^−1^. Figure 10 a–d shows the CV curves, and Figure 10e–h shows the specific capacitances of BiFe_1-x_Cr_x_O_3_ (0 ≤ x ≤ 10) electrodes. It can be clearly seen in Figure 10a–d that all the studied electrodes showed redox peaks in the CV curve due to the transitions among the different valance states. It was found that the current response of BiFe_1−x_Cr_x_O_3_ (0 ≤ x ≤ 10) electrodes increases with an increase in scan rate. Moreover, the height of the peaks observed in the CV curve shifted towards higher potential with an increasing scan rate from 10 to 100 mV s^−1^. The specific capacitance (see Figure 10e–h) determined using the CV plot was observed to decrease with the increase in scan rate. Kotz et al. [47] reported that this type of behavior results due to the presence of inner active sites, which prevent the redox transitions in the CV at higher scan rates [14]. It can be clearly seen in Figure 10e–h that the electrodes of all the compositions exhibited the maximum value of specific capacitance of 10 mV s^−1^. Figure 10a highlights the comparison of CV plots of all the compositions at the scan rate of 10 mV s^−1^. It is noteworthy to mention here that 5%-Cr-doped BiFeO_3_ (see Figure 11b) has the maximum area under the curve in the CV plot, which demonstrates the highest capacitive reaction compared to other Cr-doped BiFeO_3_-based electrodes. Figure 11b represents the comparison of the specific capacitance with different Cr doping levels for BiFeO_3_ measured at the scan rate of 10 mV s^−1^. The values of the specific capacitance calculated using the CV curve at the scan rate of 10 mV s^−1^ were 196.64, 337.0, 421.32 and 234.47 F g^−1^ for pure, 1%, 5% and 10%-Cr-doped BiFeO_3_, respectively. Moreover, we observed that with the increase in scan rate from 10 to 100 mV s^−1^, the specific capacitance decreased (see Figure 11b) from ~421 to ~200 F g^−1^, respectively. In the past, many groups have studied the electrochemical performances of BiFeO_3_-based electrodes. Lokhande et al. studied the electrochemical performances of BiFeO_3_ thin films, and they found a specific capacitance of 81.0 F g^−1^ [2]. Likewise, 72.0 F g^−^^1^ of specific capacitance has been reported for BiFeO_3_ nanoflakes by Jadhav et al. [8]. BiFeO_3_ nanorods have also shown a specific capacitance ~450 F g^−^^1^ [15,48]. Even Cu-doped BiFeO_3_ structures have also shown a specific capacitance of 568.13 F g^−^^1^, as reported by Khajonrit et al. [14]. Since the stability of the electrode is very important for technological applications, the cyclic performance of 5%-Cr-doped BiFeO_3_ was tested for 2000 cycles, as shown in Figure 11c. It was found that the capacity retention of 5%-Cr-doped BiFeO_3_ was ~78%, which represents notable cyclic stability. Kinetics and interfacial behaviors of the fabricated electrodes of 5%-Cr-doped BiFeO_3_ were measured using electrochemical impedance spectroscopy (EIS). Figure 11d shows EIS spectra of the electrode before and after the performance of 2000 cycles. In the EIS spectrum, the x-intercept of the curve in the high frequency region replicates the equivalent series resistance (ESR), which arises due to the resistance originating from numerous sources, including the internal resistance of electrode material, that of the electrolyte and the resistance at the interface [49]. Figure 11d shows the value of ESR of 1.23 Ω for the 5%-Cr-doped BiFeO_3_ electrode before the cycling test. After 2000 cycles, a slight increase in the ESR value of about 2.70 Ω was observed. Furthermore, no distinct semicircle was observed in EIS spectra, which implies that the 5%-Cr-doped BiFeO_3_ electrode exhibits excellent capacitive behavior [50,51].

## 4. Conclusions

BiFe_1−x_Cr_x_O_3,_ (0 ≤ x ≤ 10) nanoparticles were prepared via sol–gel technique. The synthesized nanoparticles were characterized using various techniques, viz., X-ray diffraction, high-resolution field emission scanning electron microscopy (HRFESEM), energy dispersive spectroscopy (EDS), UV–Vis absorption spectroscopy, photoluminescence (PL) and dc magnetization, to investigate the structural, morphological, optical and magnetic properties. The Rietveld refinement revealed the presence of secondary phases (Bi_25_FeO_39_) along with the rhombohedral (R3c) primary phase BiFeO_3_. The crystallite sizes and lattice parameter were found to decrease with increasing Cr concentration, accompanied by an enhancement in strain. The HRFESEM micrographs and EDS spectra revealed spherical morphology. Bi, Fe, O and Cr had major contributions in the samples. The UV–Vis spectra revealed a reduction in the bandgap from 1.91 to 1.74 eV with the change (increase) in Cr concentration. The investigation of PL spectra revealed emissions in violet, blue and green regions. The magnetic field (H)-dependent magnetization (M) indicated significant effects of Cr substitution on the magnetic properties of the nanoparticles. The ferromagnetic character of the samples increased with the increase in Cr concentration, and saturation magnetization increased as well. The NEXAFS analysis revealed the Fe (+3/+4) to be dissolved in mixed-valence states. Electrochemical measurements of Cr-doped BiFeO_3_ nanoparticles imply that 5%-Cr-doped BFO showed the highest value of specific capacitance: 421 F g^−1^, measured with a scan rate of 10 mV s^−1^. It also demonstrated notable cyclic stability through capacitance retention of >78% for 2000 cycles.

## Figures and Tables

**Table 1:** Density of the compound and phase fraction obtained after Rietveld refinement.

Samples	*R_p_*	*R_wp_*	*R_exp_*	*χ^2^*	Density of the Compound (g/cm^3^)	Phase Fraction (%) R 3 c/I 2 3
**BiFeO_3_**	6.55	8.81	5.48	2.54	7.862	83.15/16.85
**BiFe_0.99_Cr_0.01_O_3_**	6.74	8.70	5.46	2.54	7.857	84.96/15.04
**BiFe_0.95_Cr_0.05_O_3_**	6.96	8.80	5.29	2.77	7.733	92.72/7.28
**BiFe_0.90_Cr_0.10_O_3_**	6.56	8.55	5.37	2.54	7.684	94.99/ 5.01

**Table 2:** The values of the parameters obtained by plotting the CIE 1931 chromaticity diagram.

Samples	x	y	CCT	CRI	LER
**BiFeO_3_**	0.3561	0.354	4749	88	239
**BiFe_0.99_Cr_0.01_O_3_**	0.3581	0.3856	4696	89	234
**BiFe_0.95_Cr_0.05_O_3_**	0.3603	0.3878	4636	90	230
**BiFe_0.90_Cr_0.10_O_3_**	0.3527	0.3788	4835	89	229

## Data Availability

Not applicable.
